# Cases Illustrating the Practical Use of the Opthalmoscope, as a Means of Diagnosis in Certain Diseases of the Eye

**Published:** 1859-05

**Authors:** E. L. Holmes

**Affiliations:** Chicago, Ill.


ARTICLE II.

### CASES ILLUSTRATING THE PRACTICAL USE OF THE OPHTHALMOSCOPE, AS A MEANS OF DIAGNOSIS, IN CERTAIN DISEASES OF THE EYE.

BY E. L. HOLMES, M.D., OF CHICAGO, ILL.

[Continued from page 220.]

*Case VI. Circumscribed Retinal Infiltration.*—Patient was a young man, of delicate constitution, twenty-two years of age, whose eyes I had frequent opportunities of examining, through the kindness of his physician, a friend of mine. This young man, although so near-sighted, that, in reading, he was obliged to bring the book almost to his nose, was following a course of study in the scientific school of the city in which he lived.

His eyes becoming more easily fatigued after using them in this way, he employed a pair of powerful magnifying glasses. He soon required those of a higher power, and at last, was obliged to resort to a single convex lens (magnifying ten times), which he held, while reading, before the left eye, the right being kept closed. He continued this practice for six months, which gradually produced such an effect, that he was able to read but a few moments without causing conjunctival congestion, pain and heat in and around the eyes, with increased secretion of tears. Patient finally became alarmed, and sought the advice of a physician. Even at this time, after a little rest, the eyes appeared, externally, perfectly normal, with the exception of a trifling congestion of the conjunctivæ. The left eye was found to be much more near-sighted even than the right.

On examining the retina of the former with the ophthalmoscope, it was observed to be normal, except in the immediate vicinity of the optic nerve (papule). This last was changed from its normal, yellowish color to a light red, except a small portion of its outer half. To the inner (nasal) side of the nerve, was a very bright yellow deposit, surrounding two-thirds of its circumference, and extending in all directions from it. This was so thick that it completely covered the vessels of the retina where they passed through it.

In descriptions of this kind, it must be remembered, that the papule of the optic nerve is not, perhaps, more than a line in diameter; the arteries and veins are exceedingly fine, scarcely so large as those of the healthy conjunctiva bulbi. The deposition of which I have just spoken, could not be more than three-tenths of an inch long, and one-tenth wide, embracing the nasal side of the nerve. Now, in examining the posterior portions of an eye with reflected light, we look through the crystalline lens of the patient, which magnifies the objects behind it about twenty-four times, unless its power be modified by the lens of the ophthalmoscope. Viewed through this lens, the papule appears about an inch, or perhaps a little less, in diameter, and the arteries and veins proportionately large. In the case before us, the deposit assumed a distinct form, irregular in outline, two or more inches across its longest, and two-thirds of an inch across its shortest diameter.

There is every reason to believe, that this abnormal product, from the manner in which it covers the retinal vessels, is a deposit of plastic lymph, involving the whole thickness of the substance of the retina within the limits in which it is found.

In the right eye, the optic papule, as well as the portions of the retina, in its immediate vicinity, is highly congested, although no lymph has yet been deposited.

Undoubtedly, in the beginning, gentle antiphlogistic treatment, with due care in using the eyes, and a proper selection of glasses (if they had been found really necessary), would have prevented in a measure, at least, the progress of the disease.

Remarks.—The preceding cases, although simple, and perhaps of comparatively little interest in themselves, illustrate the use of the ophthalmoscope in establishing the diagnosis of a numerous class of ophthalmic disease.

It is true, its ability has been more than questioned by oculists of considerable reputation. It has been styled a very pretty "scientific toy," without practical use.

But, any one who is familiar with the history of ophthalmic science during the past six or eight years, must observe the increasing estimation with which the instrument is regarded, not only in Europe, but also in this country. In the *New York Journal of Medicine* (March, 1859) Dr. F. J. Bumstead, assistant surgeon of the "New York Eye and Ear Infirmary," in his report of cases, states that the ophthalmoscope "is in constant use in the Infirmary," and "is regarded as an instrument of great value, which could not now be dispensed with."

In examining cases of the slightest capsular or lenticular opacity; of effusion of blood into, or formation of floating bodies in the vitreous humor; of the numerous diseases of the retina, choroid and optic nerve, the assistance of the ophthalmoscope is invaluable. Abnormal conditions, which formerly could only be suspected, now become objects of perception, as readily *seen* as a perforation of the membrana tympani, or a polypus in the meatus.

We are aware that, unfortunately in very many cases, in which the instrument is of assistance in making our diagnosis, the disease is utterly incurable. But, even this discovery, however unpleasant it may be to the humane physician, and however painful to the afflicted patient, is better far than *doubt*, for the physician may save himself the mortification of attempting to accomplish what he can never perform; and the patient may escape a useless, and perhaps painful and *expensive* course of treatment. In medical science, and especially in diagnosis, the *fact*, the *truth*, should be the first object sought by the true physician, for this is the foundation upon which all real progress rests, and upon which every physician should base his advice and treatment.

The practical application of the instrument, and we may say the same of the microscope and stethoscope, is sometimes attended with difficulties; phenomena are occasionally observed, which cannot be interpreted upon any known principles of science. But probably these difficulties will gradually be removed, as experience shall furnish sufficient facts in the pathology of ophthalmic disease. Even with these difficulties in the way, there is scarcely an instrument, which, in its especial sphere, is able to assist the medical observer to such an extent as the ophthalmoscope.

Objections have been raised to the use of the instrument, as being injurious to the patient. They are, however, wholly groundless. Undoubtedly, too much light reflected even into a healthy eye, and especially into one affected by a disease accompanied with photophobia, would naturally tend to increase irritation. But it must be remembered that in a very large majority of instances in which the ophthalmoscope is required, the patient can bear, without trouble, the necessary degree of light, which need not exceed that of a gas light, or even the flame of a common candle. When the eye is peculiarly sensitive to light, a plain glass reflector, without amalgam, will throw sufficient light into the eye, without causing discomfort to the patient. When due regard is paid to the amount of light employed, and when the examination is made as rapidly as possible, there can never be danger of ill effects arising from it. I have seen very many patients examined with the ophthalmoscope, and have examined many myself, without ever having seen one who complained of injury.

It may be sometimes necessary to dilate the pupil with atropine. This almost always facilitates an examination, since without it the light from the reflector so contracts the pupil that it often becomes difficult to obtain a distinct image of the internal portions of the eye. The influences of atropine upon the eye is perfectly harmless, although the continued dilatation of the pupil, by admitting more light than usual, may be attended with a little inconvenience to the patient for a day or two. It is always well, in such cases, to inform the patient of what he may expect in order that he may be prepared, if necessary, to leave his ordinary employment for a couple of days.

But, fortunately, it is not in cases of grave disease alone that the ophthalmoscope may lend us valuable aid. There are a large number of people in society who are constantly injuring their eyes in an almost endless variety of ways. Even before the patient experiences trouble enough to induce him to seek the advice of a physician, there is a congestion of the retina and choroid, which can be detected with the ophthalmoscope, as is proved by those who have systematically examined a large number of individuals employed in occupations which are trying to the eyes. Many of these individuals go through life without serious inconvenience, and yet are always more or less troubled with their eyes. Others, less fortunate, perhaps being obliged to continue in occupations which tend to render their condition worse, are finally compelled to seek other means of obtaining a subsistence. The physician, who can examine such patients *early*, can be guided by the revelations of the ophthalmoscope in giving the patient rational and beneficial advice. Experience proves, that many, who follow advice thus given, have been greatly benefited.

Even in some of the graver, non-malignant diseases, dependent upon inflammatory action, the physician may be able to recognize the commencing process, long before he could do so without the ophthalmoscope. Treatment may arrest the progress of the disease which, without it, might result in plastic exudation, a termination almost always fatal to vision.

